# Three-dimensional imaging evaluation of facial swelling after orthognathic surgery with compression and Kinesio taping therapy: a randomized clinical trial

**DOI:** 10.1097/MS9.0000000000001719

**Published:** 2024-01-15

**Authors:** Hiroaki Nakao, Shogo Hasegawa, Mai Tomimatsu, Jun Sasaki, Satoshi Yamamoto, Satoshi Watanabe, Satoru Miyabe, Fumitaka Terasawa, Hitoshi Miyachi, Mitsuo Goto

**Affiliations:** Department of Maxillofacial Surgery, School of Dentistry, Aichi Gakuin University Graduate School of Medicine, Nagoya, Japan

**Keywords:** Body surface temperature, compression taping, compression therapy, kinesio tape, orthognathic surgery, postoperative swelling

## Abstract

**Background:**

Postoperative swelling is a common complication of orthognathic surgery. The authors used three-dimensional (3D) image analysis and body surface temperature to determine the effects of compression taping (CT) and Kinesio taping (KT) by the epidermis, dermis, and fascia method (EDF-KT) on postoperative swelling.

**Materials and methods:**

The authors conducted a prospective, parallel-group, randomized controlled trial. Among the 162 patients diagnosed with jaw deformity and who underwent orthognathic surgery from August 2020 to October 2022, 105 patients (men: 36, women: 69, mean age: 28.27±8.92) underwent Le Fort type I + sagittal split ramus osteotomy (SSRO) or SSRO and were included in this study. Patients were randomly divided into three groups: EDF-KT group (*n*=31), CT group (*n*=41), and no tape group (control group, *n*=30). All taping was performed immediately postoperatively and removed on postoperative day (POD) 5. Three-dimensional images of the participants’ faces were obtained preoperatively and at PODs 3, 7, 30, and 90 using a hand-held 3D imaging system and infrared thermography.

**Results:**

No significant difference was observed in postoperative swelling and postoperative body surface temperature between the groups at each time point. The CT group showed a trend towards reduced swelling on PODs 3 and 7 and a trend toward residual swelling on POD 90. The EDF-KT group showed a trend towards an increase in postoperative body surface temperature.

**Conclusion:**

CT taping may not be appropriate for postoperative swelling control, suggesting that EDF-KT may affect body surface temperature. Further validation of the efficacy of KT for jaw deformities is needed.

## Introduction

Highlights

- Postoperative swelling is a common complication of orthognathic surgery.
- Kinesio tape has been investigated as a treatment for postoperative swelling.
- The evaluation of Kinesio taping using epidermal, dermal, and fascial methods for postoperative swelling did not show reduction in postoperative swelling.

In orthognathic surgeries, extensive surgical interventions on soft and hard tissues in the craniofacial region result in postoperative swelling in the cheek and neck owing to excessive accumulation of blood, exudate, and lymphatic fluid from the damaged tissue. The risk factors for swelling include the extent of intraoperative incision and dissection and the duration of surgery. Recent studies have suggested that factor XIII deficiency is associated with exacerbated swelling and delayed wound healing^1,2^. Facial swelling after orthognathic surgery appears immediately postoperatively, commonly increases until the fourth day, and gradually disappears thereafter. Facial swelling in the immediate postoperative period can cause life-threatening complications such as airway obstruction and other complications such as inferior alveolar nerve dysfunction, pain, infection, and aesthetic concerns. Compression for postoperative swelling has been reported to be effective with compression garments^3^ in the surgical field and compression bandages^4^ in the orthopaedic field.

A report^5^ comparing facial compression band and intramuscular dexamethasone injection after wisdom tooth extraction found the facial compression band was equally effective as a single dose of dexamethasone at 48 h and 7 days postoperatively. The facial compression band induced a massaging effect through opening movements and improved lymphatic drainage. In addition, a facial compression band is reported to reduce postoperative swelling effectively.

The Kinesio Tape Association reports that Kinesio taping (KT) normalizes muscle function, promotes the repair of damaged muscles, lifts the epithelium and dermis, widens the gap between subcutaneous tissues, and improves circulation of blood and lymphatic fluid, thereby reducing swelling and pain.

In recent years, KT treatment has been reported to reduce postoperative swelling after artificial knee joint replacement surgery in orthopaedic surgeries^6^ and reduce swelling and pain after tooth extraction in dental surgeries^7,8^.

In this study, we investigated the effects of KT and computed tomography (CT) on postoperative swelling after jaw deformity surgery using three-dimensional (3D) image analysis and body surface temperature.

## Materials and methods

### Patients

The target number of cases was set at 39 for each group, based on a power analysis of the three groups with a mean difference of −0.5 to 0.5, a significance level of 5%, and a detection rate of 69.82%.

**Figure 1:**
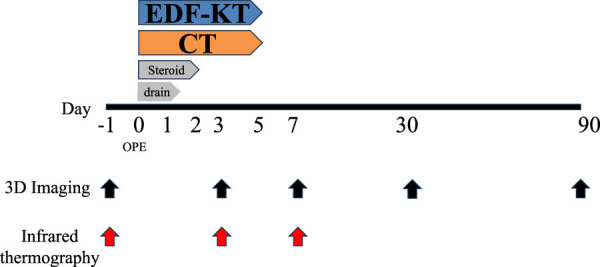
Allocation flowchart. CT, computed tomography; EDF-KT, epidermis, dermis, and fascia method-Kinesio taping.

Among the 162 patients diagnosed with jaw deformities and who underwent orthognathic surgery between August 2020 and October 2022 at the Department of Maxillofacial Surgery, School of Dentistry, Aichi-Gakuin University, 105 patients (men: 36, women: 69) were included, excluding those with bleeding predispositions, postoperative factor XIII deficiency, or postoperative infection and those who underwent Le Fort 1 only or genioplasty. Written informed consent was obtained from all patients prior to study participation. Surgery was performed using Le Fort I osteotomy for the maxilla and bilateral sagittal split ramus osteotomy (SSRO) for the mandible. All surgeries were performed by three maxillofacial surgeons skilled in orthognathic surgery. Perioperative steroids included 4 mg of betamethasone intraoperatively and on postoperative day (POD) 1 and 2 mg on POD 2. A closed suction drain was inserted at the surgical site of the mandible and removed on POD 1 (Fig. 1).

This study was approved by the Ethics Committee of Aichi-Gakuin University (Approval No. 556). All procedures of this study were conducted in accordance with the principles of the Declaration of Helsinki, “Ethical Principles for Medical Research Involving Human Subjects.”

### Allocation

**Figure 2:**
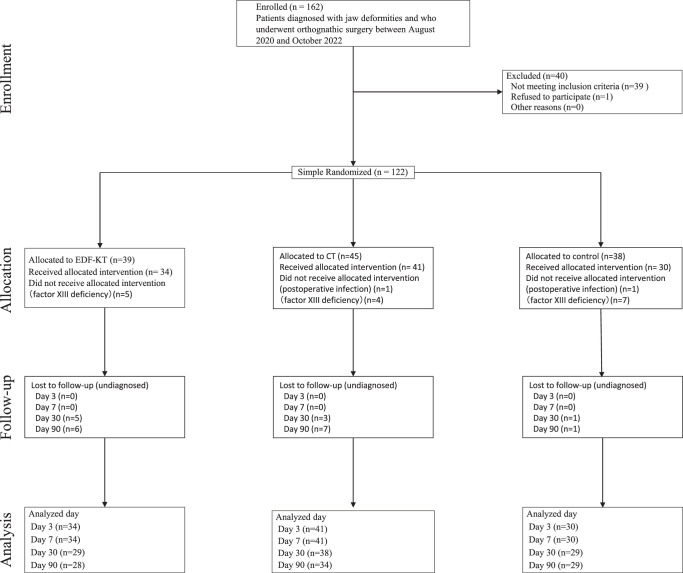
Research progress. CT, computed tomography; EDF-KT, epidermis, dermis, and fascia method-Kinesio taping.

Patients were divided by simple randomization into three groups: KT group using the epidermis, dermis, fascia (EDF) taping technique method (EDF-KT group), CT group, and control group. A flowchart showing the number of subjects in each phase of the randomized controlled trial is shown in Fig. 2. A simple, blinded, prospective, parallel-group, randomized comparative study was conducted and reported according to the CONSORT 2010 statement.

The protocol for this study is registered with the University Hospital Medical Information Network (UMIN) Center. (Clinical Trial Registration Number: UMIN000052484) (URL:https://www.umin.ac.jp/ctr/index.htm).

### Taping method

**Figure 3:**
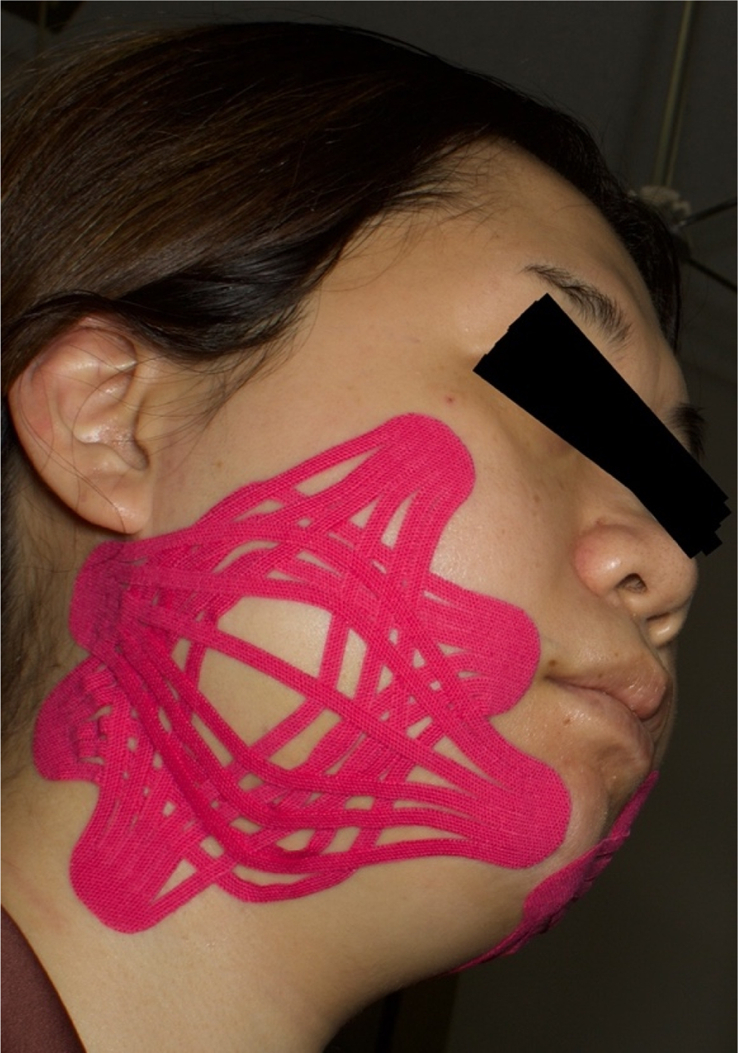
Kinesio taping using the epidermis, dermis, and fascia method.

Three sheets of Kinesio Tex Gold FP PRE-CUT (150 mm long × 25 mm wide, with slits) were used for the EDF-KT technique after a discussion with the instructor of the Kinesio Tape Association on the most effective method of applying KT for swelling after jaw deformity surgery. The first tape was applied from the neck to the corner of the mouth through the angle of the mandible. The second tape was applied from the submandibular area to the posterior auricular region, and the third tape was taped from the zygomatic arch to the pharynx, forming a hexagon at the centre with the other two tapes, with a tension of 5–10% (Fig. 3), (Table 1). All applications were performed by the same researcher with a KT certification.

**Table 1:** Kinesio taping method

	First tape	Second tape	Third tape
Target organization	Fascia, subcutaneous tissue	Dermis	Epidermis
Taping method	From the neck to the corners of the mouth	From under the chin to the auricle Apply with the application site developed.	Apply from the zygomatic bone to the mandible angle (along the masseter muscle). With the application site extended, apply the first and second tapes to the centre of the tape to form a hexagon.
Tension	5–10%	5–10%	5–10%
Purpose of adhesion	Reduction of oedema and heat sensation. Promotes circulation of interstitial fluid.	Reduction of swelling and heat sensation. Promotes circulation of blood and lymph.	Pain relief (severe pain)

**Figure 4:**
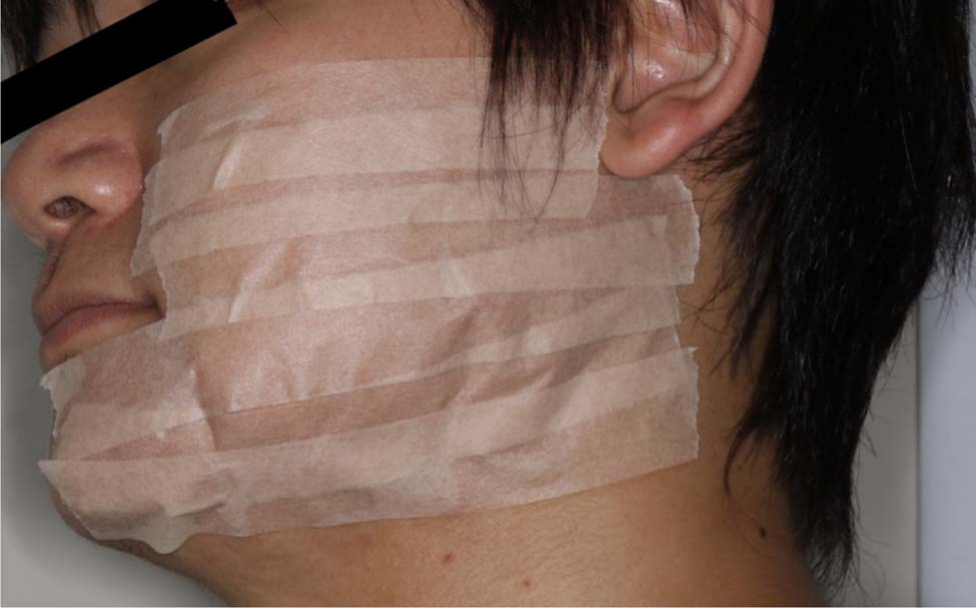
Compression tape.

The CT method was applied using a 25 mm wide YU-KI-BAN (Nitto), a surgical tape for medical use to cover the maximum swelling area and allow for compression (Fig. 4). CT was performed by a dentist familiar with the technique.

Both KT and CT were applied immediately postoperatively and removed on POD 5. The taping was performed after removing sufficient moisture and fat from the skin surface. If the taping spontaneously detached during the taping period, a new tape was immediately reapplied.

### Measurement methods

**Figure 5:**
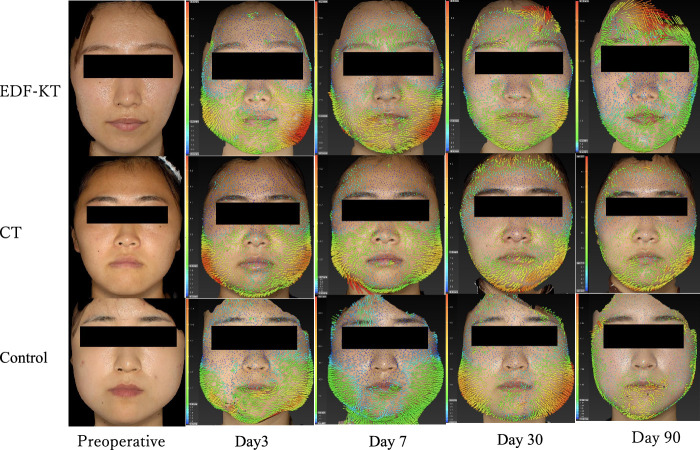
Superimposition and swelling measurement before surgery. CT, computed tomography; EDF-KT, epidermis, dermis, and fascia method-Kinesio taping.

The swelling was measured using the VECTRA H1 (Canfield Scientific Inc), a non-contact 3D imaging analyzer. Images were obtained preoperatively and at PODs 3, 7, 30, and 90. The frontal images were obtained at a distance where the two laser alignment points intersected at a single point in the philtrum. Lateral views were taken at 45° obliquely downward at a distance where the laser alignment intersected a point midway between the zygomatic bone and the zygomatic arch. The preoperative and postoperative data were superimposed using the automatic analysis function of the 3D construction software Vectra (Canfield Scientific Inc) version (2022_10_1300_33_39), and the swelling volume (cm^3^) was measured (Fig. 5). All data were analyzed using STL data.

**Figure 6:**
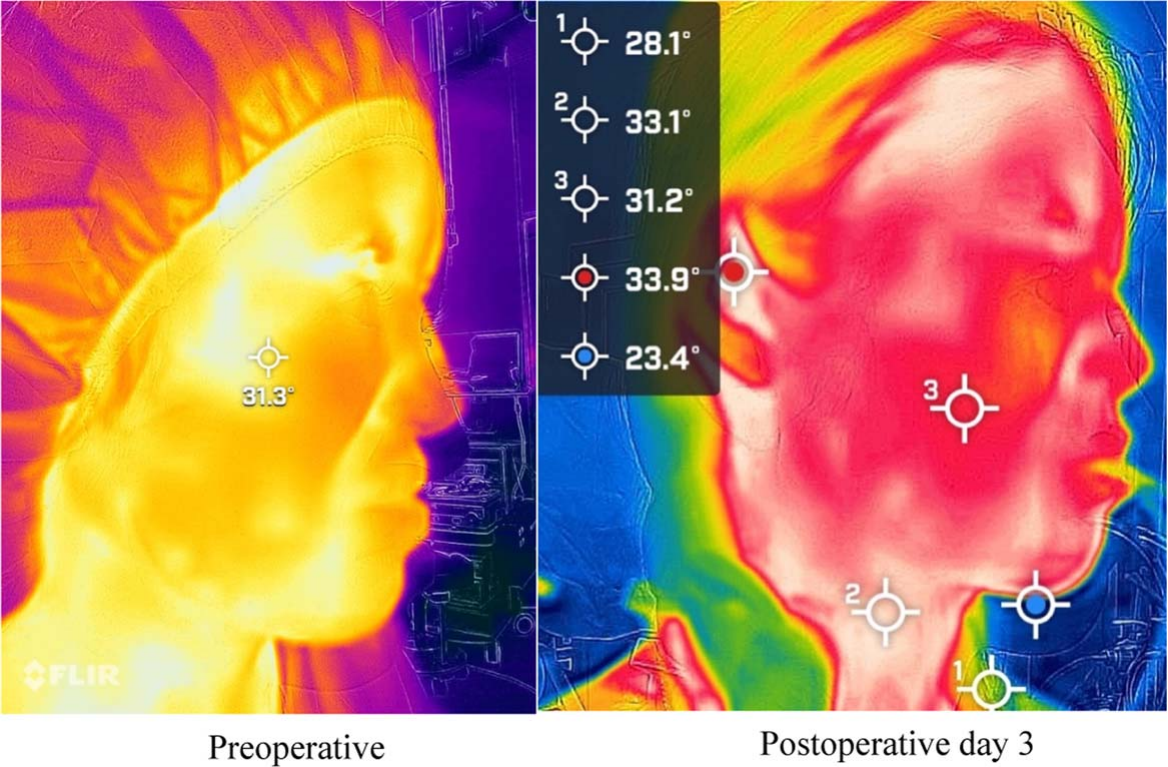
Infrared thermography imaging.

The body surface temperature measurements were obtained using a FLIR ONE Pro infrared thermography camera (FLIR), with a thermal image resolution of 1.55 megapixels at 60 ×120 pixels and an accuracy of ± 5%. Furthermore, it is inexpensive and can be used as a thermal imaging camera when connected to a smartphone. The maximum body surface temperatures (°C) of the bilateral buccal swellings were measured during hospitalization (preoperatively and POD 3 and 7) to minimize the differences in ambient temperature that could affect body surface temperature measurements. Body surface temperatures were not measured in the CT group because taping precluded this measurement (Fig. 6).

All imaging was performed in the sitting position to eliminate changes in body surface temperature due to postural changes in volume and blood flow. All imaging was performed under fluorescent lighting in the same treatment room in the hospital ward using a dental care chair.

### Statistical methods

The Shapiro–Wilk test was used to test for normality of the swelling volume and body surface temperature.

Nonparametric tests were the Kruskal–Wallis test for swelling volume and the Wilcoxon test for body surface temperatures. For swelling volume, subgroup analysis was performed for surgical procedures.

Statistical analysis was performed using the JMP software version 17; SAS Institute, Cary). Statistical significance was set at *P* less than 0.05.

## Results

The mean patient age was 25 (22–31) years, BMI was 20.14 (18.37–21.98), and mean operative time was 137 (114–251) h for SSRO alone and 239.5 (198.75–266.25) h for SSRO + Le Fort I. Intraoperative blood loss was 76.5 (47.5–142.5) ml for SSRO alone and 146.5 (92.25–255) ml for SSRO + Le Fort I (Table 1) (Table 2). The Kruskal–Wallis test showed no significant difference in postoperative swelling between the groups at each time point (Table 3). Subgroup analysis by surgical technique also showed no significant differences between the groups at any point in time (Tables 4 and 5). The amount of facial swelling did not follow a normal distribution on PODs 3 and 7. The Wilcoxon test for postoperative body surface temperature showed no significant differences between the groups at any point in time (Table 6).

**Table 2:** Target details

	EDF-KT	CT	Control	*P*
Patients (persons)	34	41	30	
Gender ratio (m/f)	10/24	14/27	12/18	
Average age median (IQR) age	26 (22–32.25)	25.5 (21–35)	26 (21–31)	0.81
Le Fort 1+sagittal split ramus osteotomy	18	20	18	
Sagittal split ramus osteotomy	16	21	12	
Operation time median (IQR) min	211 (130.75–256.25)	208 (116.75–252)	242.5 (149–281)	0.14
Bleeding volume median (IQR) ml	125 (67.5–181.75)	100 (57.5–180)	130 (86.5–295)	0.15

CT, computed tomography; EDF-KT, epidermis, dermis, and fascia method-Kinesio taping; f, female; IQR, interquartile range; m, male.

**Table 3:** Comparison of swelling volume at postoperative days 3, 7, 30, and 90

	EDF-KT	CT	Control	*P*
Day 3	52.14 (37.93–62.80)	44.27 (30.12–49.67)	50.45 (37.92–56.68)	0.10
Median (IQR)	(*n*=34)	（*n*=41）	(*n*=30)	
Day 7	41.77 (31.43–55.47)	33.29 (20.76–40.69)	38.93 (22.18–50.46)	0.10
Median (IQR)	(*n*=34)	(*n*=41)	(*n*=30)	
Day 30	16.22 (9.48–21.66)	13.81 (7.45–21.38)	12.43 (8.78–22.53)	0.88
Median (IQR)	(*n*=29)	(*n*=38)	(*n*=29)	
Day 90	1.49 (0.44–3.45)	3.05 (1.34–6.94)	2.15 (1.28–3.45)	0.48
Median (IQR)	(*n*=28)	(*n*=34)	(*n*=29)	

CT, computed tomography; EDF-KT, epidermis, dermis, and fascia method-Kinesio taping; IQR, interquartile range.

**Table 4:** Results of subgroup analysis according to surgical technique (sagittal split ramus osteotomy)

	EDF-KT	CT	Control	*P*
Day 3	50.38 (36.11–67.13)	42.57 (31.33–47.62)	50.38 (31.13–58.56)	0.30
Median (IQR)	(*n*=16)	(*n*=21)	(*n*=12)	
Day 7	42.14 (24.62–55.56)	30.34 (19.87–38.74)	39.47 (21.11–50.78)	0.20
Median (IQR)	(*n*=16)	(*n*=21)	(*n*=12)	
Day 30	14.14 (8.64–22.11)	13.84 (5.07–21.91)	18.22 (8.23–28.88)	0.80
Median (IQR)	(*n*=15)	(*n*=19)	(*n*=11)	
Day 90	2.48 (1.49–4.11)	3.75 (1.85–6.21)	1.90 (1.85–6.21)	0.37
Median (IQR)	(*n*=14)	(*n*=16)	(*n*=11)	

CT, computed tomography; EDF-KT, epidermis, dermis, and fascia method-Kinesio taping; IQR, interquartile range.

**Table 5:** Results of subgroup analysis according to surgical technique (Le Fort I + sagittal split ramus osteotomy)

	EDF-KT	CT	Control	*P*
Day 3	52.14 (39.67–59.06)	46.22 (29.43–56.60)	50.74 (42.10–56.80)	0.52
Median (IQR)	(*n*=18)	(*n*=20)	(*n*=18)	
Day 7	41.74 (31.65–52.33)	35.66 (22.60–44.22)	36.93 (22.63–51.19)	0.57
Median (IQR)	(*n*=18)	(*n*=20)	(*n*=18)	
Day 30	17.18 (9.01–21.14)	13.79 (9.27–21.22)	11.88 (9.15–20.45)	0.78
Median (IQR)	(*n*=14)	(*n*=19)	(*n*=18)	
Day 90	2.38 (1.33–3.45)	2.62 (1.15–7.45)	2.30 (1.13–3.45)	0,97
Median (IQR)	(*n*=14)	(*n*=18)	(*n*=18)	

CT, computed tomography; EDF-KT, epidermis, dermis, and fascia method-Kinesio taping; IQR, interquartile range.

**Table 6:** Comparison of body surface temperature

	EDF-KT (*n*=34)	Control (*n*=27)	*P*
Day 3 median (IQR)	31.90 (30.04–33.71)	31.01 (30.06–32.90)	0.20
Day 7 median (IQR)	31.53 (29.91–33.19)	30.52 (30.01–32.9)	0.32

EDF-KT, epidermis, dermis, and fascia method-Kinesio taping; IQR, interquartile range.

## Discussion

Several treatment methods have been devised to reduce postoperative swelling after orthognathic surgery. In this study, we evaluated the effects of KT and CT on postoperative swelling after jaw deformity surgery and the effect of KT on body surface temperature. We hypothesized that EDF-KT and CT would reduce swelling and increase body surface temperature in the early postoperative period. However, no significant difference was observed in postoperative facial swelling in the EDF-KT or CT groups compared to the controls. No change was observed in the body surface temperature between the EDF-KT and control groups.

Treatments for postoperative swelling include cooling^9^, manual lymphatic drainage^10^, steroids^11–13^ proteolytic enzymes^14,15^, and laser therapy^16^; however, some of these techniques are inconvenient and require special equipment and methods.

Steroid administration suppresses phospholipase A2 and the subsequent production of inflammatory mediators and is effective in suppressing postoperative swelling and pain. Postoperative steroid administration is considered safe^17^. However, the use of steroids is not entirely free of side effects^18^.

KT requires no special equipment or technology, is unlikely to cause side effects, and is highly convenient. KT has contractility similar to that of human muscles and raises the subcutaneous tissue by ~10 μm when performed correctly, improving the local circulation of blood and lymphatic fluid and reducing swelling and pain. KT is commonly applied in the fields of sports medicine, rehabilitation, and orthopaedic surgery. KT reduces postoperative oedema after breast cancer surgery^19,20^ and knee arthroplasty^21^.

Most studies that have examined the effectiveness of Kinesio taping after tooth extraction and orthognathic surgery have used a taping technique called the Lymphatic Kinesio Taping Technique^7,8,22,23^. In this method, a 50 mm wide tape was divided into four equal strips to the base after orthognathic surgery, with the undivided end of the tape applied to the supraclavicular tuberosity and the fourth equal strip applied slightly below the height of the zygomatic arch. The tape was applied using the lymphatic KT method, in which KT is applied across the cervical, submandibular, submandibular, preauricular, and parotid lymph nodes and directed toward the lymphatic vessels to reach the zygomatic arch. Swelling improved from the fifth day post-application and disappeared within 10 days, demonstrating KT’s efficacy in reducing postoperative swelling.

To date, there have been no reports on the application of EDF-KT after orthodontic jaw surgery.

The EDF-KT is a taping method believed to be more effective than lymphatic KT in improving blood and interstitial fluid circulation in the applied area and suppressing postoperative pain. However, its effectiveness in suppressing postoperative swelling after orthognathic surgery yielded negative results. The inference was made that variations in results could be influenced by factors such as the application method, including the position and shape of the tape.

**Figure 7:**
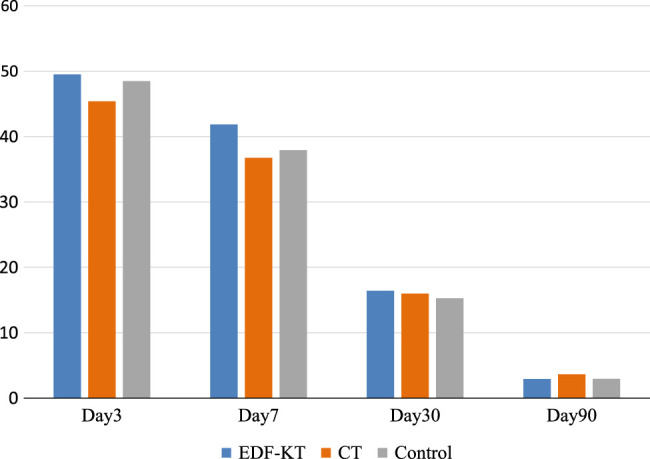
The CT group showed a trend towards decreased postoperative swelling compared to the KT and control groups on PODs 3 and 7. However, residual swelling is observed at POD90. CT, computed tomography; EDF-KT, epidermis, dermis, and fascia method-Kinesio taping.

CT is a taping method devised at our hospital for reducing postoperative swelling after orthognathic surgery, based on the suppression effect of postoperative oedema by compression garments in the fields of surgery, obstetrics and gynaecology, and orthopaedics^3,4^. This is the first report on the effects of CT and KT after orthognathic surgery using a long-term follow-up study. No statistically significant differences were observed among the three groups. However, the CT group showed a trend towards decreased postoperative swelling compared to the KT and control groups on PODs 3 and 7. This result is similar to a report comparing facial compression bandages and intramuscular dexamethasone injection^5^, suggesting that CT has a swelling suppression effect on PODs 3 and 7 (Fig. 7).

However, long-term residual swelling may occur after CT owing to impaired blood flow circulation from the pressure on the wound. Furthermore, the effects of CT and KT are expected to vary depending on the tape position and shape. KT and CT require further verification of their effects, including verification of differences from controls.

Furthermore, the relationship between the effect of EDF-KT on body surface temperature was investigated. No change in body surface temperature was observed postoperatively, and no change was observed after KT application. Severe postoperative oedema after axillary dissection for breast cancer is reported to decrease body surface temperature due to impaired blood flow^24^. An increase in body surface temperature was expected after EDF-KT; however, no statistically significant differences were found. Nevertheless, the EDF-KT group showed a trend towards an increase in body surface temperature compared to the CT group, possibly owing to improvement in blood and lymphatic flow.

In addition, the measurement results of thermography cameras are affected by ambient temperatures. Therefore, this study, which was conducted over a long time period, may have been influenced by seasonal changes in ambient temperature.

Methods for measuring facial swelling include facial surface topography, photographic methods, and MRI^25,26^. Facial surface topography is extremely complex and does not adequately reflect the extent of facial swelling. In addition, conventional soft tissue volume imaging techniques, such as MRI and cone-beam computed tomography (CBCT), are difficult to use in research and clinical settings owing to cost and radiation exposure^27,28^. Therefore, the use of non-contact 3D imaging analysis devices is increasing. This technique uses lasers and charge-coupled device (CCD) cameras (digital) to enable an accurate and objective 3D evaluation of facial morphology. The VECTRA H1 non-contact 3D image capture and analysis device used in this study is a CCD camera-type device that is radiation-free, portable, quick, and capable of capturing accurate and highly reproducible stereophotogrammetric images^29–31^, with an error of 0.20 ml. The virtual 3D model can accurately detect small volume changes; therefore, we concluded that errors due to the measurement equipment did not affect this study.

### Limitations

Our sample size was small, and we did not account for factors related to postoperative swelling, such as surgical difficulty, which may not be evenly distributed among the groups.

Based on the results of this study, a total sample size of 273 cases, with 91 cases per group, was determined using a significance level (α) of 0.05, the standard deviation and mean observed on the seventh postoperative day, and a statistical power of 0.8.

All surgeries were performed by several skilled physicians, and patients with postoperative factor XIII deficiency were excluded, mitigating any interoperator effects.

The possibility that swelling volume and body surface temperature were affected by seasonal climatic variations cannot be excluded.

## Conclusion

No differences in postoperative swelling were observed for KT or CT compared to controls, and KT did not affect body surface temperature. CT showed a tendency for long-term residual swelling and may not be appropriate for postoperative swelling control. EDF-KT showed a trend for increased body surface temperature, and the effectiveness of KT in jaw deformities requires further verification.

## Ethical approval

Ethics approval for this study (approval number: 556) was granted by the Ethics Committee of Aichi Gakuin University in Aichi, Japan on 9 April 2019.

## Consent

Written informed consent was obtained from the patient for publication of this case report and accompanying images. A copy of the written consent is available for review by the Editor-in-Chief of this journal on request.

## Source of funding

No research sponsors involved.

## Author contribution

H.N.: taping, data collection, data analysis, interpretation, writing papers. S.H.: conception or design of the study, writing of the paper, significant revisions to the paper approval of the final draft. M.T.: data collection. J.S.: Taping, data collection. S.Y.: taping, data collection. S.W.: taping, important revisions to the paper. S.M.: important revisions to the paper. F.T.: important revisions to the paper. H.M.: important revisions to the paper. M.G.: important revisions to the paper.

## Conflicts of interest disclosure

The author has received kinesio tape from the Kinesio Taping Association. This study was conducted fairly and properly, without being influenced by the interests or intentions of the donors.

## Research registration unique identifying number (UIN)

Registration: University hospital Medical Information Network (UMIN) Center. Clinical Trials Registry Public title：Effect of Kinesio Taping and Compression Taping on Facial Swelling After Orthognathic Surgery URL: https://www.umin.ac.jp/ctr/index.htm UIN: UMIN000052484.

## Guarantor

Shogo Hasegaw.

## Data availability statement

Data can be disclosed upon request.

## Provenance and peer review

Not applicable.
